# An Endoparasitoid Avoids Hyperparasitism by Manipulating Immobile Host Herbivore to Modify Host Plant Morphology

**DOI:** 10.1371/journal.pone.0102508

**Published:** 2014-07-17

**Authors:** Tomohisa Fujii, Kazunori Matsuo, Yoshihisa Abe, Junichi Yukawa, Makoto Tokuda

**Affiliations:** 1 Biosystematics Laboratory, Graduate School of Social and Cultural Studies, Kyushu University, Fukuoka, Japan; 2 Japan Society for the Promotion of Science, Chiyoda-ku, Tokyo, Japan; 3 Entomological Laboratory, Graduate School of Bioresource and Bioenvironmental Sciences, Kyushu University, Fukuoka, Japan; 4 Entomological Laboratory, Faculty of Agriculture, Kyushu University, Fukuoka, Japan; 5 Laboratory of System Ecology, Faculty of Agriculture, Saga University, Saga, Japan; University of Tours, France

## Abstract

Many parasitic organisms have an ability to manipulate their hosts to increase their own fitness. In parasitoids, behavioral changes of mobile hosts to avoid or protect against predation and hyperparasitism have been intensively studied, but host manipulation by parasitoids associated with endophytic or immobile hosts has seldom been investigated. We examined the interactions between a gall inducer *Masakimyia pustulae* (Diptera: Cecidomyiidae) and its parasitoids. This gall midge induces dimorphic leaf galls, thick and thin types, on *Euonymus japonicus* (Celastraceae). *Platygaster* sp. was the most common primary parasitoid of *M. pustulae*. In galls attacked by *Platygaster* sp., whole gall thickness as well as thicknesses of upper and lower gall wall was significantly larger than unparasitized galls, regardless of the gall types, in many localities. In addition, localities and tree individuals significantly affected the thickness of gall. Galls attacked by *Platygaster* sp. were seldom hyperparasitized in the two gall types. These results strongly suggest that *Platygaster* sp. manipulates the host plant's development to avoid hyperparasitism by thickening galls.

## Introduction

Many parasites and parasitoids manipulate their hosts to improve their own fitness and performance. Such manipulation includes biochemical, physiological, and behavioral changes of their hosts [1], [2], [3]. Parasites often facilitate their own transmission by altering host behavior [4], [5], [6]. Some insect parasitoids are known to change their host's behavior to enhance their own fitness by reducing the risk of predation or hyperparasitism [7], [8], [9]. For example, aphids parasitized by *Aphidius nigripes* Ashmead (Hymenoptera: Braconidae) leave the colony and hide in concealed microhabitats to avoid the attack of hyperparasitoids [10], [11]. Larvae of *Pieris brassicae* (L.) (Lepidoptera: Pieridae) parasitized by a primary parasitoid, *Cotesia glomerata* (L.) (Hymenoptera: Braconidae), change their behavior after *C. glomerata* larvae quit the host larvae. The larvae protect cocoons of *C. glomerata* against hyperparasitoids by spinning silk webs over the cocoons [12], [13]. Larvae of *Thyrinteina leucocerae* Rindge (Lepidoptera: Geometridae) parasitized by a braconid wasp *Glyptapanteles* sp. defend cocoons of the parasitoid against approaching predators with violent head swinging [14], [15].

Up to the present, studies of host manipulation by insect parasitoids have focused mainly on those associated with mobile hosts but seldom on those associated with endophytic, immobile, or sessile hosts. Primary parasitoids of immobile hosts, such as gall inducers, cannot move with the hosts to less dangerous sites to avoid predation and hyperparasitism. However, the avoidance of hyperparasitism is very important because the rate of hyperparasitism often becomes very high [e.g. 16, 17, 18]. For gall inducers, thicker gall walls are advantageous because they prevent oviposition by late parasitoids (see reference [16] for the definition of ‘early and late parasitoid’; see the Materials and Methods) after maturation of galls [e.g. 20, 21, 22, 23]. A previous study proposed that primary parasitoids might manipulate their hosts to modify gall traits, e.g. thickness of gall wall and hardness of gall tissue, to avoid hyperparasitism [23].

In this paper, we focus on the euonymus gall midge *Masakimyia pustulae* Yukawa & Sunose, 1976 (Diptera: Cecidomyiidae) and its primary parasitoid, a platygastrid species (Hymenoptera). By examining the relationship between gall thickness and percentage parasitism by the platygastrid and late parasitoids, we tested the hypothesis that the primary parasitoid manipulates host larvae to induce increased gall thickness to avoid hyperparasitism.

## Materials and Methods

### Biology of *Masakimyia pustulae*

**Figure 1:**
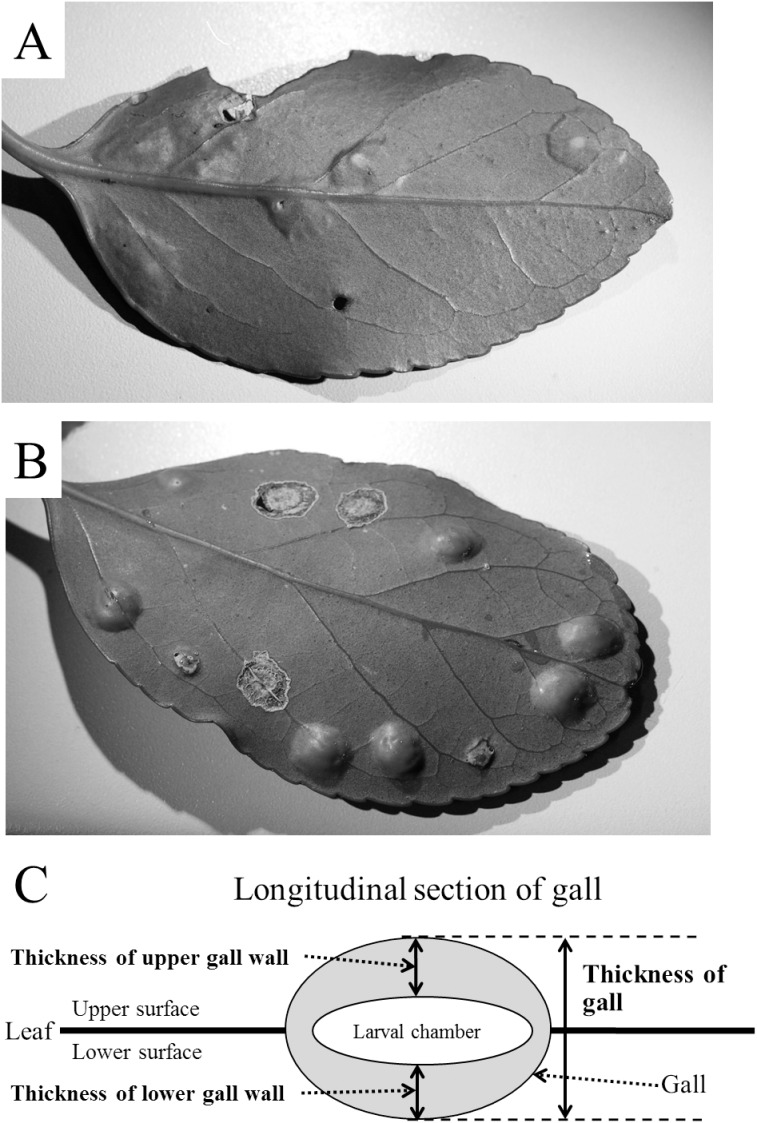
Blister-like leaf galls induced by *Masakimyia pustulae* on *Euonymus japonicus*. A: thin type gall, B: thick type gall, C: schematic illustration showing the method of measuring gall-wall thickness.

*Masakimyia pustulae* is univoltine and is present in Japan [24] and Korea [25]. The gall midge induces dimorphic leaf galls, both thick and thin types (Fig. 1), on *Euonymus japonicus* Thunb. and *E. fortunei* Hand-Mazz (Celastraceae) [26]. The thick type of gall is slightly swollen on the upper surface and subovate on the lower surface of the host leaf. The thin type of gall is a flat and very weak swelling on both surfaces of the host leaf. The thickness of gall ranges from 1.4 to 2.8 mm in the thick type and from 0.7 to 1.3 mm in the thin type, respectively [24]. Galls are single-chambered and each contains one gall midge larva [26]. Females of *M. pustulae* usually lay their eggs on the lower surface of fresh host leaves in spring and rarely on 2 or 3 year-old leaves. Immediately after hatching, first instars go into the host leaf tissue and molt into second instars before summer. They become third ( = final) instars in autumn, and pupation takes place in the following spring.

The galls grow rapidly when the larvae are in the third instars and mature in size in the following spring [24], [27]. The adults emerge in southern Kyushu in early March and in northern Kyushu in late March. As in other gall-inducing cecidomyiids, the adult life of *M. pustulae* is very short, approximately 12 hours in males and mated females, and about 24 hours in unmated females [27].

**Figure 2:**
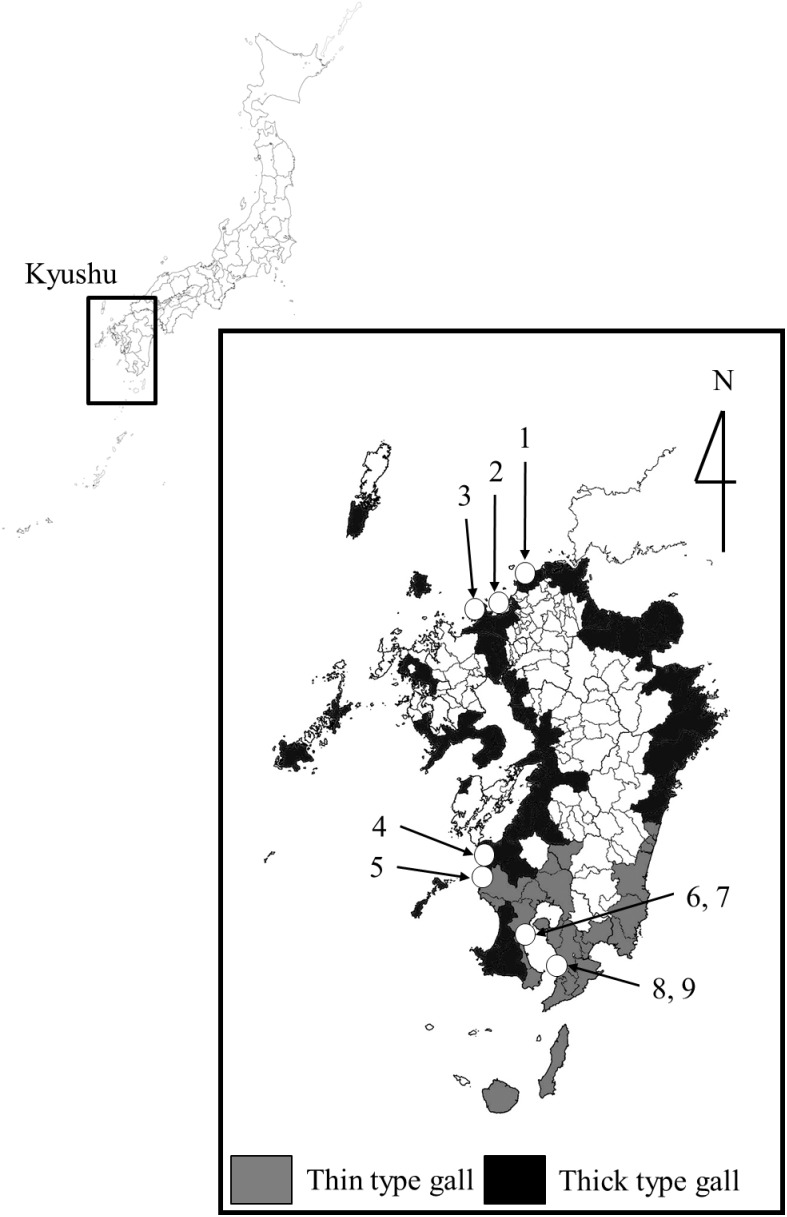
Distribution of thick and thin types of gall induced by *Masakimyia pustulae* on *Euonymus japonicus* Thunb. in Kyushu, Japan (modified from Sunose, 1985a). Numerals indicate localities where galls were collected (see also Table 1).

The gall midge population inducing thick type galls and that inducing thin type galls are distributed almost parapatrically in Japan [27]. The former is found in the northeast areas of Honshu (Tohoku District), southern parts of Shikoku, and southern parts of Kyushu (Fig. 2). The latter is widely found in other areas of Honshu, Shikoku and Kyushu (Fig. 2). Crossing experiments between the two gall midge populations suggested that quantitative genes of the gall midge are probably responsible for the dimorphism in gall shape [27]. Populations of intermediate type galls are often found in the distributional boundaries between both gall types [27].

### Parasitoids associated with *Masakimyia pustulae*

Parasitoid wasps of gall inducers (e.g. gall wasps and gall midges) are divided into two groups according to their parasitic strategies [16], [28], [29], [30]. Early parasitoids are usually host-specific, univoltine, and koinobiont endoparasitoids, which allow hosts to continue to grow, ovipositing into eggs or first instars of hosts before the development of the gall to its full size. Late parasitoids are generally polyphagous, multivoltine, and idiobiont ectoparasitoids (rarely endoparasitoids), which kill or permanently paralyze their host, attacking older hosts, such as final instars or pupae, after the galls mature by inserting long ovipositors through the gall wall. When both early and late parasitoids attack the same host larva, only the latter idiobiont species can survive [19], [27], [31]. Thus late parasitoids are regarded as facultative hyperparasitoids attacking both the unparasitized gall midge larvae and those already parasitized by early parasitoids [19], [27].

Eight species of solitary parasitoids consisting of five eulophids, one pteromalid, one braconid and one platygastrid are parasitic on *M. pustulae* [19], [27]. The platygastrid is a koinobiont early parasitoid ovipositing into eggs of *M. pustulae*. The larva of this parasitoid starts to grow rapidly after *M. pustulae* larvae have developed to the final instar, pupates inside the host body in late winter, and the adult emerges in early spring [19]. At the time of pupation, the host integument enclosing the platygastrid becomes hard and turns brown. The other seven species are idiobiont late parasitoids attacking the final instars or pupae of *M. pustulae* from autumn to the following spring [19]. They are *Chrysocharis sunosei* Kamijo, *Chrysonotomyia* sp., *Pnigalio* sp., *Tetrastichus* sp. A, *Tetrastichus* sp. B (Eulophidae), *Spaniopus japonicas* Kamijio (Pteromalidae) and *Bracon tamabae* Maetô (Braconidae). Among the late parasitoids, three species of eulophids, *Chrysonotomyia* sp., *Tetrastichus* sp. A and sp. B, and the braconid are ectoparasitoids, while the pteromalid and the other two eulophids are endoparasitoids.

### Collection, measurements and dissection of galls

Host leaves with *M. pustulae* galls were collected from nine localities in Kyushu in February and March 2009, 2010 and 2012 (Table 1). In 2009, 10 to 50 galled leaves (depending on the size of tree) were randomly collected from a single host tree in each locality. In 2010, one or two trees were selected in each locality to examine differences in gall thickness between trees. In Munakata, only one host tree was available for sampling. Then, five current shoots with galled leaves were randomly collected from each tree. After measuring gall thickness with digital slide calipers (Fig. 1), galls were dissected under a stereoscopic microscope to examine inhabitants to record their developmental stage. In 2012, two and nine shoots with galled leaves were collected from a single host tree in Munakata and Tenjin, respectively, to measure upper and lower gall wall thicknesses under a stereoscopic microscope with an ocular micrometer to confirm whether or not the increase in gall thickness is related to the thickness of both upper and lower gall walls.

**Table 1:** Collecting sites of galls induced by *Masakimyia pustulae* in Kyushu, Japan.

No.	Collecting site	Location		Collecting date			Gall type
		N	E	2009	2010	2012	
1	Munakata, Fukuoka	33°53′16″	130°31′31″	9 Mar.	21 Mar.	6 Mar.	Thin
2	Shikanoshima, Fukuoka	33°39′51″	130°18′50″	9 Mar.	21 Mar.		Thin
3	Itoshima, Fukuoka	33°38′24″	130°12′02″	10 Mar.	22 Mar.		Thin
4	Nishikata, Kagoshima	31°54′39″	130°13′27″	21 Feb.	13 Mar.		Thin
5	Satsumataki, Kagoshima	31°53′21″	130°13′21″	21 Feb.	13 Mar.		Thin
6	Meiwa, Kagoshima	31°36′06″	130°31′20″	11 Feb.	14 Feb.		Thick
7	Sanwa, Kagoshima	31°33′04″	130°33′18″	17 Feb.	12 Feb.		Thick
8	Tenjin, Kagoshima	31°22′46″	130°46′40″	25 Feb.	14 Feb.	24 Mar.	Thick
9	Takasu, Kagoshima	31°20′44″	130°47′42″	25 Feb.	14 Feb.		Thick

### Parasitoids and percentage parasitism

Parasitoids were identified using Sunose [19]. Percentage parasitism was calculated for each parasitoid species in each locality. In particular, a parasitoid pupated inside the skin of full-grown host larva was identified as one of the platygastrid species. The gall thickness was compared between galls containing host larvae attacked by the platygastrid (parasitized galls, hereafter) and those with unparasitized larvae (unparasitized galls, hereafter). The thicknesses of upper and lower gall walls were also compared between galls parasitized by the platygastrid and unparasitized galls. The percentage parasitism by late parasitoids was compared between galls with or without the platygastrid. In this case, platygastrids attacked by late parasitoids were recognized by the presence of late parasitoid larvae or pupae in the galls, together with brownish fragments of cecidomyiid larval integument that had been sclerotized by the platygastrid. To examine whether late parasitoids preferably attack inhabitants in thinner galls, the thickness of gall was compared between galls attacked by late parasitoids and those that were not.

### Statistical analyses

Statistical analyses were performed using R 2.12.1 [32]. Differences in the thickness between galls of unparasitized hosts and those parasitized by the platygastrid or late parasitoids were analyzed using two-way ANOVA for the 2009 data. The mean values were compared between inhabitants in each tree by Student's t-test using *p* values adjusted by Holm’s method [33] to reduce the family wide error rate. Nested ANOVA was used for comparing the thickness between galls of unparasitized hosts and those parasitized by the platygastrid, in which tree individuals are nested within localities and inhabitants (unparasitized hosts or those parasitized by parasitoids) are nested within tree individuals within localities. When significant effects of individual trees were detected, the mean values were compared between inhabitants in each census tree by Student's t-test using *p* values adjusted by the Holm’s method. In case the sample size was less than three in any category, they were excluded from the analysis. Differences in the thickness of upper and lower gall walls between parasitized and unparasitized galls were compared using one-way ANOVA. Percentage parasitism by late parasitoids between galls with and without the platygastrid was compared with chi-square test or paired Fisher’s exact test when the minimum expected value was less than 5.0, using *p* values adjusted by the Holm’s method. Nested ANOVA was used for comparing the thickness between galls of unparasitized hosts and those parasitized by the late parasitoids. When significant effects of individual trees were detected, the comparison of mean values between inhabitants in each tree by Student's t-test using *p* values adjusted by the Holm’s method.

## Results

### Percentage parasitism by early and late parasitoids

We obtained three of eight known parasitoids of *M. pustulae* through the current surveys in Kyushu. They were an early parasitoid, platygastrid and two species of late parasitoids, *Pnigalio* sp. and *Chrysonotomyia* sp. One of us, KM identified the platygastrid as *Platygaster* sp. based on a literature [34].

*Platygaster* sp. was found in most localities surveyed and its percentage parasitism was higher in many areas than that by *Pnigalio* sp. and *Chrysonotomyia* sp., except in some localities (Tables 2 & 3). Either *Pnigalio* sp. or *Chrysonotomyia* sp. exhibited relatively high percentage parasitism in Shikanoshima in 2009, on Tree 1 and 3 in Shikanoshima in 2010. In particular, percentage parasitism by the late parasitoids exceeded 50% on Tree 1 in Nishikata and on Trees 1 and 2 in Satsumataki in 2010.

**Table 2:** Percentage parasitism by early and late parasitoids in galls induced by *Masakimyia pustulae* in 2009.

Census field	n	MP	Early parasitoid	Late parasitoids	
			PL	CH	PN
Munakata	217	28.1	68.7	3.2	0.0
Shikanoshima	102	52.0	16.7	0.0	31.3
Itoshima	118	83.1	16.7	0.0	0.8
Nishikata	31	74.2	22.6	3.2	0.0
Satsumataki	133	27.8	23.3	36.1	12.8
Meiwa	177	86.4	13.6	0.0	0.0
Sanwa	376	37.8	62.0	0.0	0.3
Tenjin	184	32.1	67.9	0.0	0.0
Takasu	12	25.0	66.7	0.0	8.3

Abbreviations are as follow: n: the number of galls examined, MP: unparasitized larvae and pupae of *Masakimyia pustulae*, PL: *Platygaster* sp., CH: *Chrysotonomyia* sp. and PN: *Pnigalio* sp.

**Table 3:** Percentage parasitism by early and late parasitoids in galls induced by *Masakimyia pustulae* in 2010.

Census field	Tree	N	MP	Early parasitoid	Late parasitoids	
				PL	CH	PN
Munakata	T_1_	192	40.6	46.4	6.3	6.8
Shikanoshima	T_1_	110	23.6	55.5	6.4	14.5
	T_2_	85	18.8	74.1	2.4	4.7
	T_3_	81	63.0	17.3	19.8	0.0
Itoshima	T_1_	119	96.6	0.0	3.4	0.0
	T_2_	237	83.5	13.1	0.0	3.4
Nishikata	T_1_	71	7.0	12.7	50.7	29.6
	T_2_	34	58.8	14.7	23.5	1.1
Satsumataki	T_1_	94	18.1	16.0	64.9	1.1
	T_2_	16	12.5	6.3	18.8	62.5
Meiwa	T_1_	37	73.0	27.0	0.0	0.0
	T_2_	64	65.6	32.8	0.0	1.6
Sanwa	T_1_	52	1.9	98.1	0.0	0.0
	T_1_	102	90.2	9.8	0.0	0.0
Tenjin	T_1_	137	72.3	27.7	0.0	0.0
	T_2_	66	48.5	50.0	0.0	1.5
Takasu	T_1_	26	73.1	23.1	0.0	0.0
	T_2_	37	59.5	40.5	0.0	0.0

Abbreviations are as follow: n: the number of galls examined, MP: unparasitized larvae and pupae of *Masakimyia pustulae*, PL: *Platygaster* sp., CH: *Chrysotonomyia* sp., PN: *Pnigalio* sp., T_1_: Tree1, T_2_: Tree 2 and T_3_: Tree 3.

### Comparison in thickness between galls unparasitized and parasitized by *Platygaster* sp

**Figure 3:**
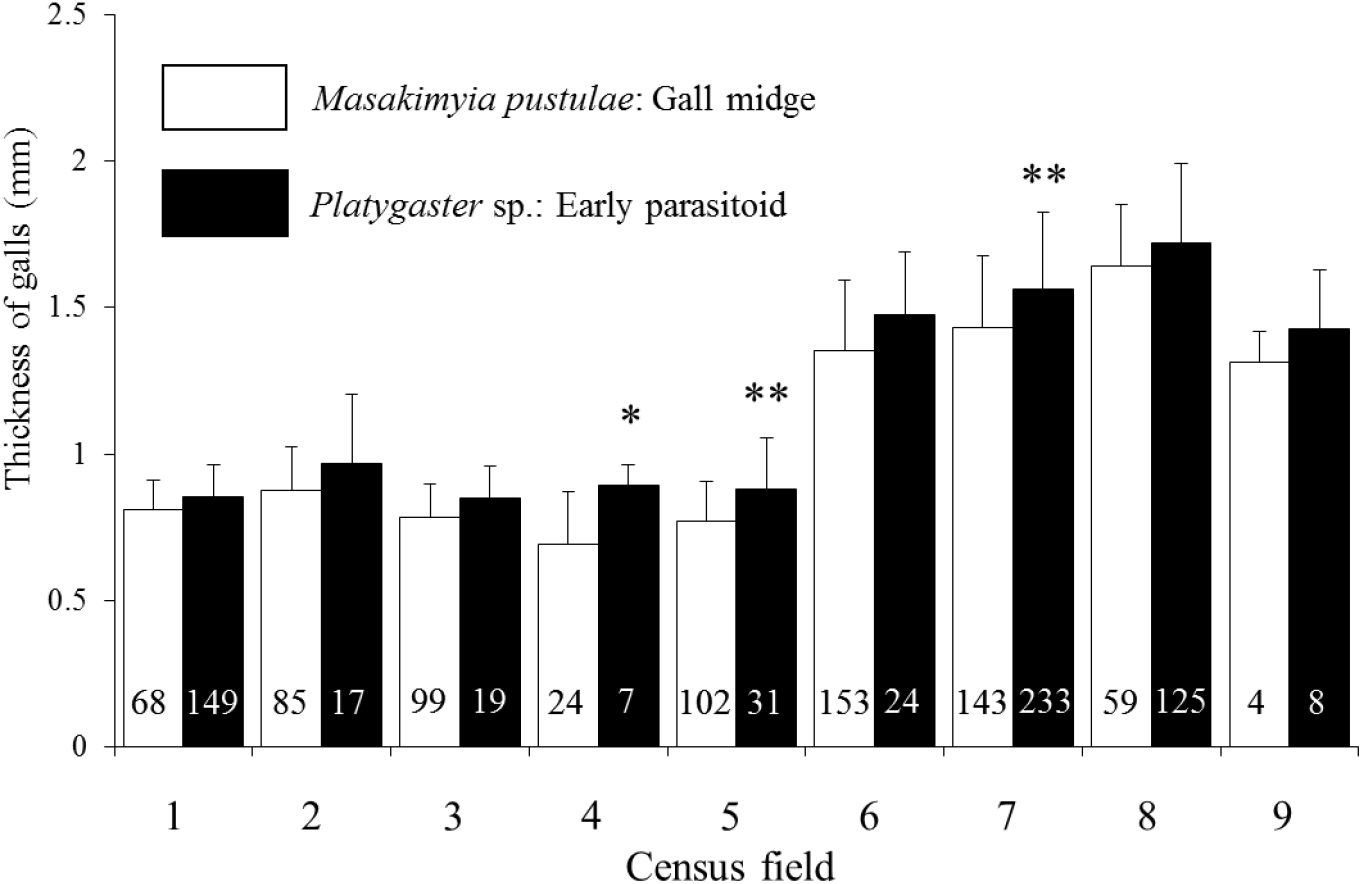
The thickness of galls containing unparasitized *Masakimyia pustulae* and those parasitized by *Platygaster* sp. in 2009. 1: Munakata, 2: Shikanoshima, 3: Itoshima, 4: Nishikata, 5: Satsumataki, 6: Meiwa, 7: Sanwa, 8: Tenjin, 9: Takasu. The number of galls examined was shown in each column. Asterisk indicates significant differences at *5% level or **1% level (two-way ANOVA followed by Student’s t-test in each tree with adjusted *p* values by Holm’s method).

**Figure 4:**
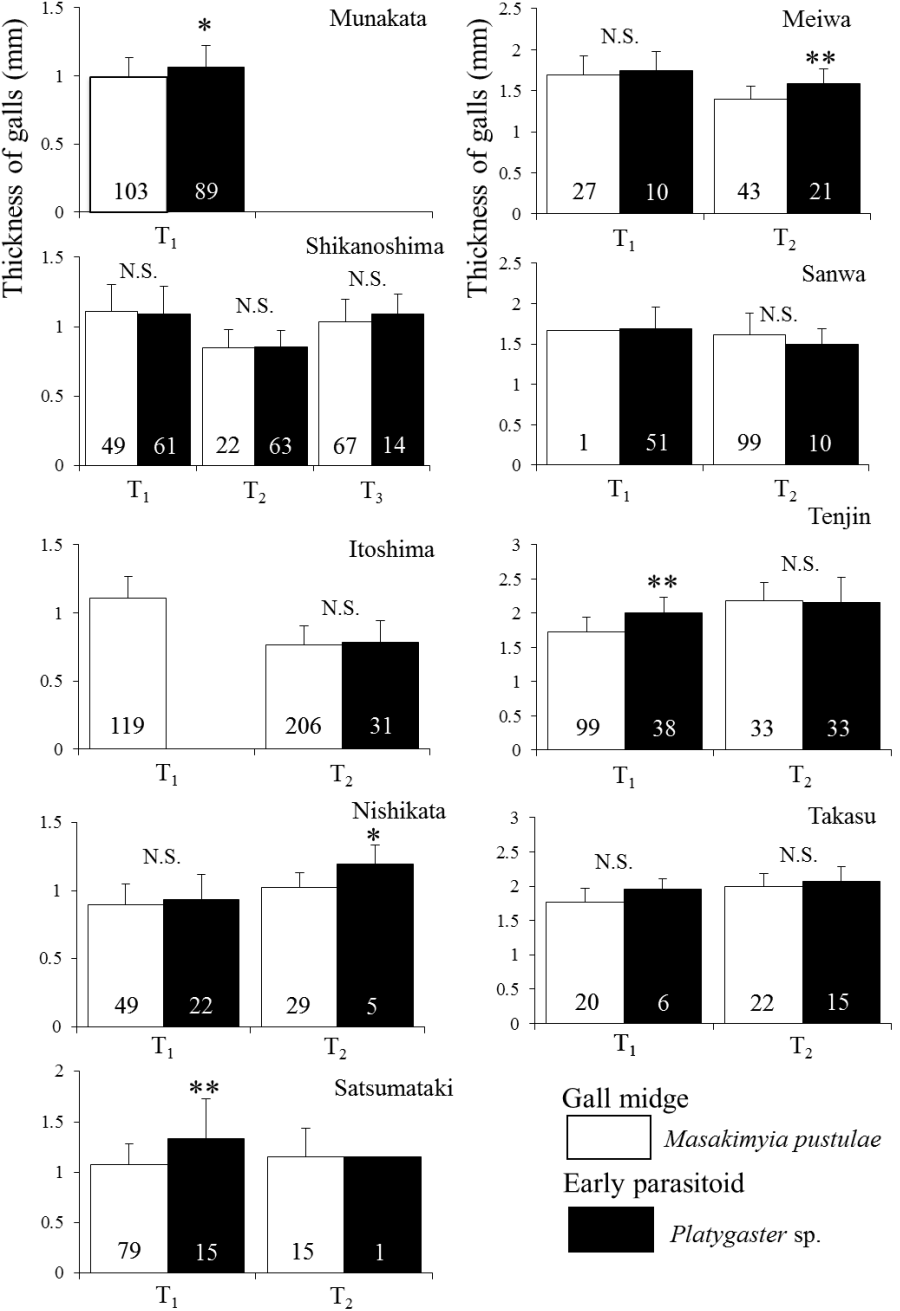
The thickness of galls containing unparasitized *Masakimyia pustulae* and those parasitized by *Platygaster* sp. in 2010. The number of galls examined was shown in each column. Sample size is shown in parentheses. Abbreviations are as follows, T_1_: Tree 1, T_2_: Tree 2, and T_3_: Tree 3. Asterisk indicates significant differences at 1% level (nested ANOVA followed by Student’s t-test in each tree with adjustment of *p* values by Holm’s method; asterisk indicates significant difference).

**Figure 5:**
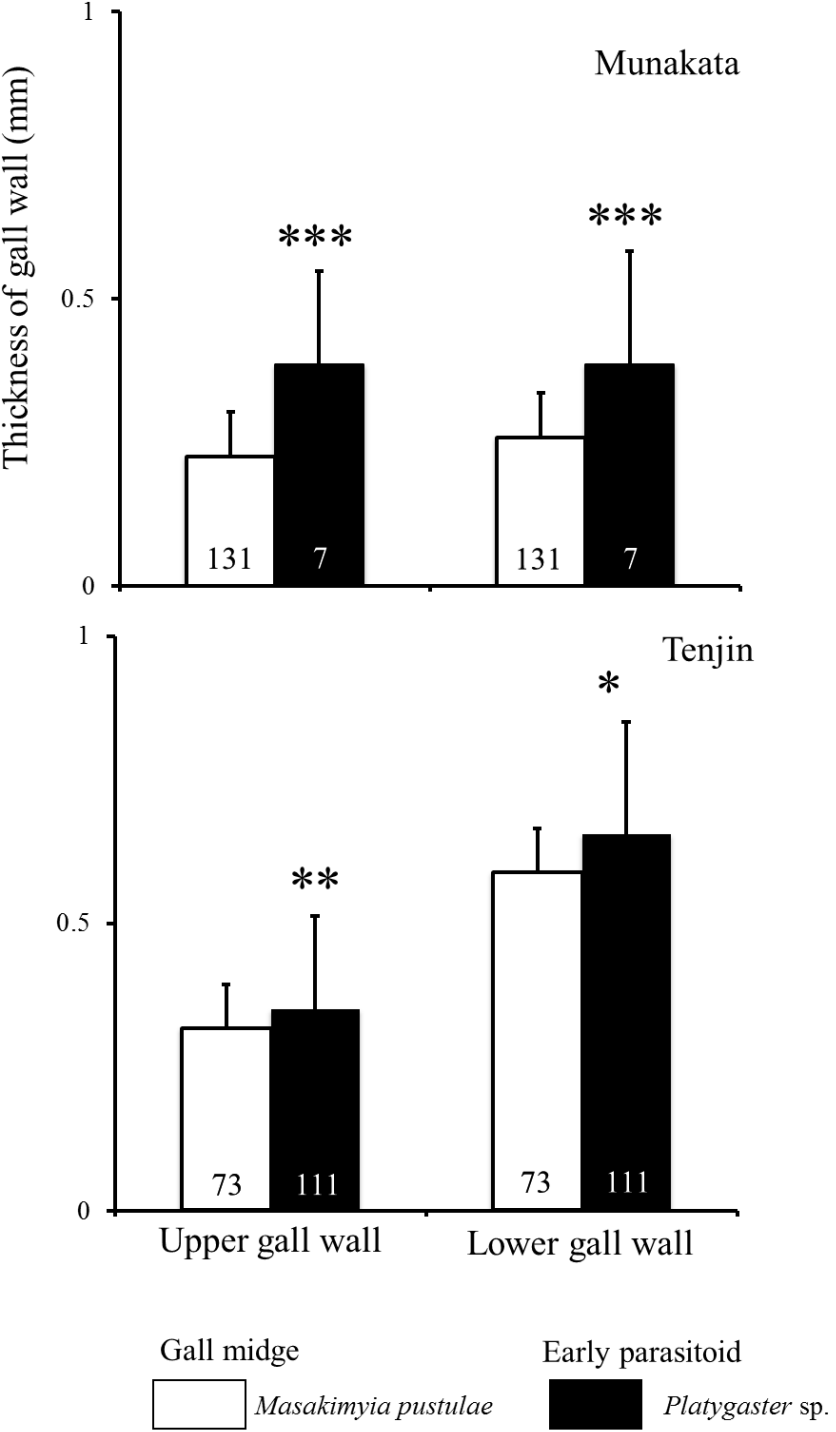
The thickness of upper and lower gall walls of unparasitized *Masakimyia pustulae* galls and those parasitized by *Platygaster* sp. in 2012. The number of galls examined was shown in each column. Asterisk indicates significant differences, *P<0.05, **P<0.01, ***P<0.001 (one-way ANOVA).

Regardless of the gall type, the thicknesses of galls parasitized by *Platygaster* sp. were significantly higher (0.1–0.5 mm) than those unparasitized in three out of the nine trees surveyed in 2009 (Fig. 3). In 2010, the thickness was significantly different between unparasitized and parasitized galls on four out of the 18 trees surveyed in five out of nine localities, but not significantly different on the remaining trees and localities (Fig. 4; see Table 4 for the result of nested ANOVA). Based on the survey in 2012, both upper and lower gall walls were significantly thicker in galls parasitized by *Platygaster* sp. than those unparasitized (Fig. 5).

**Table 4:** Results of nested ANOVA to detect the effects of the platygastrid, tree individual and locality on the thickness of gall induced by *Masakimyia pustulae.*

	df	Mean Sum Square	F	*p*
*Platygaster* sp.	1	1.4477	37.9036	<0.001
*Platygaster* sp. & Tree	8	0.1883	4.3813	<0.001
*Platygaster* sp. & Locality	8	0.1673	4.9249	<0.001
Error	1525	0.0382		

### Comparison in parasitism by the late parasitoids between galls unparasitized and parasitized by *Platygaster* sp

In 2009, the late parasitoids were not found in the galls parasitized by *Platygaster* sp., and the percentage parasitism by the late parasitoids was significantly lower in the galls parasitized by *Platygaster* sp. in three of five trees surveyed in 2009 (Table 5). In 2010, the parasitism by the late parasitoids in the galls parasitized by *Platygaster* sp. was found only on Tree 1 in Nishikata, where the gall thickness was not significantly different between unparasitized and parasitized galls (Fig. 4). The percentage parasitism by the late parasitoids was remarkably high (90%) in the unparasitized galls on Tree 1 in Nishikata (Table 6). The percentage parasitism by the late parasitoids was significantly lower in the galls parasitized by *Platygaster* sp. than in those unparasitized on five trees in four localities. In two of the four localities, the gall thickness was significantly different between unparasitized and parasitized galls. The percentage parasitism by the late parasitoids also tended to be lower on the remaining trees and localities regardless of the gall thickness (Table 6).

**Table 5:** Percentage parasitism by late parasitoids in galls containing only unparasitized *Masakimyia pustulae* larvae (Unparasitized MP) and in those containing host larvae parasitized by *Platygaster* sp. (Parasitized by PL) in 2009.

Census filed	Unparasitized MP		Parasitized by PL		*P*	*x^2^*
	n	% parasitism	n	% parasitism		
Munakata	68	10.3	149	0.0	<0.001	15.85
Shikanoshima	85	37.6	17	0.0	<0.01	9.326
Itoshima	99	1.0	19	0.0	1.00	
Nishikata	24	4.2	7	0.0	1.00	
Satsumataki	102	63.7	31	0.0	<0.001	38.64

Percentage parasitism was analyzed with chi-square test for the sample from Munakata, Shikanoshima and Satsumataki, and with paired Fisher’s exact probability test for those from Itoshima and Nishikata.

**Table 6:** Percentage parasitism by late parasitoids in galls containing only unparasitized *Masakimyia pustulae* larvae (Unparasitized MP) and in those containing host larvae parasitized by *Platygaster* sp. (Parasitized by PL) in 2010.

Census field	Tree	Unparasitized MP		Parasitized by PL		Significance	*x^2^*
		n	% parasitism	n	% parasitism		
Munakata	T_1_	103	24.3	89	0.0	**	24.84
Shikanoshima	T_1_	49	46.9	61	0.0	**	36.20
	T_2_	22	27.3	63	0.0	**	
	T_3_	67	23.9	14	0.0	n. s.	
Itoshima	T_1_	119	3.4	0	0.0	n. s.	
	T_2_	206	3.9	31	0.0	n. s.	
Nishikata	T_1_	49	89.8	22	59.1	**	
	T_2_	29	31.0	5	0.0	n. s.	
Satsumataki	T_1_	79	78.5	15	0.0	**	34.58
	T_2_	15	86.7	1	0.0	n. s.	

Percentage parasitism was analyzed with chi-square test for T_1_ in Munakata, T_1_ in Shikanoshima and T_1_ in Satsumataki, and with paired Fisher’s exact probability test for other tree with adjusted *p* values by Holm’s method (significantly different at *5% or **1% level). Abbreviations are as follow: T_1_: Tree1, T_2_: Tree 2 and T_3_: Tree 3.

### Comparison in thickness between galls unparasitized and parasitized by the late parasitoids

**Figure 6:**
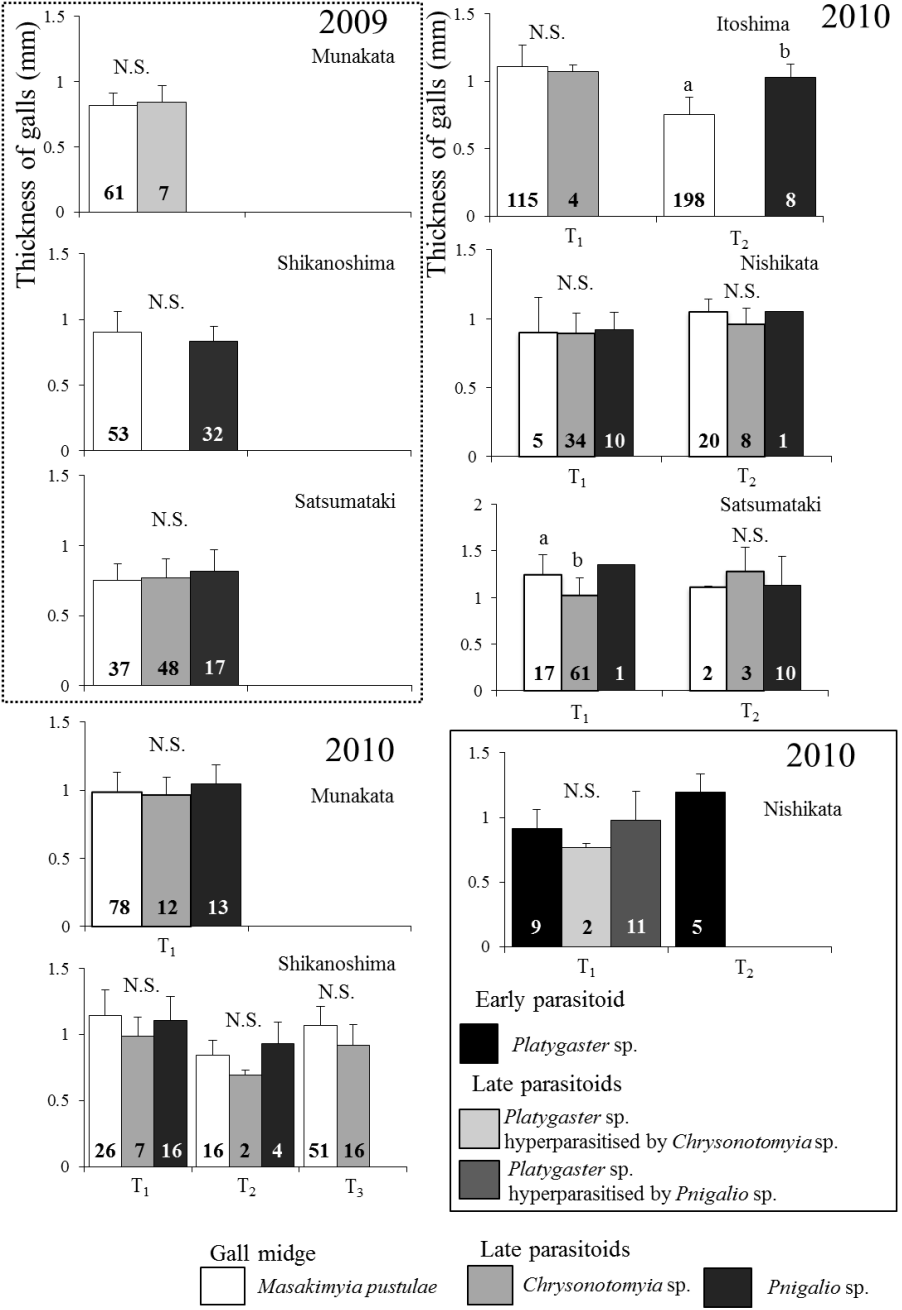
The thickness of galls containing unparasitized *Masakimyia pustulae* larvae and those parasitized by the late parasitoids. The number of galls examined was shown in each column. Abbreviations are as follows, T_1_ : Tree 1, T_2_: Tree 2, and T_3_: Tree 3. Different letters indicate significant differences at 5% level (nested ANOVA followed by pairwise comparisons of Student’s t-test in each tree with adjustment of *p* values by Holm’s method). The thickness of galls parasitized by *Platygaster* sp. and those hyperparasitized by the late parasitoids was shown at the bottom right.

Galls parasitized by *Pnigalio* sp. were significantly thinner than those unparasitized on one out of the three trees surveyed in 2009 (Fig. 6). Data of Itoshima and Nishikata in 2009 are not shown because the late parasitoids were seldom recorded (Table 2). In 2010, galls parasitized by *Chrysonotomyia* sp. were significantly thinner than those unparasitized on Tree 1 in Satsumataki (Fig. 6). Although no significant differences were detected on the remaining nine trees, five of them exhibited similar tendency to the tree (Fig. 6; see Table 7 for the result of nested ANOVA). Galls parasitized by *Pnigalio* sp. were significantly thicker than those unparasitized on Tree 2 in Itoshima, but not significantly different on the remaining trees and in other localities. The thickness of galls parasitized by *Platygaster* sp. was not significantly different between those subsequently parasitized and not parasitized by *Pnigalio* sp. (Fig. 6).

**Table 7:** Results of nested ANOVA to detect the effects of late parasitoids, tree individual and localities on the thickness of gall induced by *Masakimyia pustulae.*

	df	Mean Sum Square	F	*p*
Late parasitoids	2	0.4128	17.5875	<0.001
Late parasitoid & Tree	7	0.1053	4.4933	<0.001
Late parasitoid & Locality	8	0.0453	1.9311	0.062
Error	711	0.0235		

## Discussion

Our study clearly indicated that the hypertrophy of *M. pustulae* galls was associated with a decreased rate of parasitism by the late parasitoids. In contrast to the relatively high percentage parasitism by the early parasitoid both in the thick and thin types of gall (Tables 2 & 3), parasitism by the late parasitoids was seldom found in the thick type gall at Meiwa and Sanwa (Tables 2 & 3). On the contrary, more than 50% of galls were parasitized by the late parasitoids on Tree 1 in Nishikata and on Trees 1 and 2 in Satsumataki in 2010 (Table 3). These results strongly suggest that the hypertrophy of galls is effective in preventing parasitism by the late parasitoids, as mentioned by Sunose [26].

Galls parasitized by *Platygaster* sp. were significantly thicker than those unparasitized in most individual trees (Figs. 3 & 4). Because gall thickness was strongly affected by tree individualities (Table 4), it was not significantly different between unparasitized galls and those parasitized by *Platygaster* sp. on some trees (Fig. 4). However, galls parasitized by *Platygaster* sp. were mostly thicker than those unparasitized. Both the upper and lower gall walls were significantly thicker in galls parasitized by *Platygaster* sp. than in those unparasitized (Fig. 5). These results strongly suggest that galls parasitized by *Platygaster* sp. are adapted for avoiding hyperparasitism by the late parasitoids, because hypertrophy of gall wall is well known to decrease their parasitism rates [e.g. 31, 35] probably by preventing their ovipositors from penetrating the gall wall. Significant interaction effects of individual trees and localities were also detected (Table 4). Although the mechanism is still unknown, these results suggest that some trees and some local populations of *M. pustulae* may not respond to the manipulation by *Platygaster* sp.

The percentage parasitism by the late parasitoids was significantly lower in galls parasitized by *Platygaster* sp. than those unparasitized (Tables 5 & 6). These results strongly suggest that *Platygaster* sp. manipulates the behavior of *M. pustulae* larvae, possibly by enhancing their feeding activity, to thicken the gall wall to avoid hyperparasitism. An alternative possibility is that females of *Platygaster* sp. may be able to detect differences in host egg size, if any. They then prefer to lay their eggs in large host eggs, which are expected to give rise gall midge larvae that induce thick galls.

There were no significant differences in gall thickness between unparasitized and parasitized galls by *Platygaster* sp. on Tree 1 in Nishikata (Fig. 4), but percentage parasitism by the late parasitoids was significantly lower in galls parasitized by *Platygaster* sp. (Table 6). This suggests that *Platygaster* sp. avoids hyperparasitism by the late parasitoids not only by manipulation of the gall shape but also by other mechanisms, such as manipulation of gall wall hardness or of chemical components in gall tissue including volatiles emitted by the gall and attracting the late parasitoids. Otherwise, the late parasitoids may have an ability to recognize the existence of pupae of the early parasitoid inside the gall midge body, as has been known for some other parasitoids [e.g. 36], or may avoid attacking gall midge larvae that had been parasitized by *Platygaster* sp. Galls parasitized by *Chrysonotomyia* sp. were significantly thinner than unparasitized galls on two out of the ten trees surveyed in Shikanoshima and Satsumataki (Fig. 6). These results suggest that *Chrysonotomyia* sp. attack inhabitants in thinner galls more frequently and that *Platygaster* sp. increases their own survival rates by the hypertrophy of galls.

A recent study suggested that a braconid parasitoid *Cotesia rubecula* Marshall parasitizing *Pieris rapae* (L.) (Lepidoptera: Pieridae) decreased the survival rate of another parasitoid *C. glomerata* in different host individuals that feed on the same plant, through the manipulation of plant traits, probably plant-induced secondary metabolites [37]. However, quite a few studies have demonstrated the manipulation of plant characteristics by parasitoids to avoid the risk of predation and hyperparasitism. As far as we know, such a phenomenon was reported by a literature [23], in which the author briefly mentioned that galls induced by the gall wasp *Andricus quadrilineatus* Hartig (Hymenoptera: Cynipidae) became thicker and harder when they were attacked by an early parasitoid *Aulogymnus euedoreschus* Walker (Hymenoptera: Eulophidae). Moreover, the literature supposed that galls attacked by *A. euedoreschus* might be hardly penetrable by other parasitoids [23].

Our study demonstrated for the first time that the manipulation of plant morphology through the host insect by the early parasitoid was associated with a decreased rate of hyperparasitism by the late parasitoids. Therefore such manipulation is adaptive for the early parasitoid. Future investigations to clarify the mechanism underlying the host manipulation by the *Platygaster* sp. are necessary to understand its parasitic strategy and evolutionary process of tri-trophic interactions centered upon the immobile larvae of *M. pustulae*. Moreover, further studies focusing on immobile host–parasitoid systems may reveal various manipulation mechanisms by primary parasitoids to avoid the risk of hyperparasitism and predation.

## References

[1] Vinson SB, Iwantsch GF (1980) Host regulation by insect parasitoids. Quar Rev Biol 55(2): 143–165.

[2] Harvey JA, Kos M, Nakamatsu Y, Tanaka T, Dicke M, et al (2008) Do parasitized caterpillars protect their parasitoids from hyperparasitoids? A test of the ‘usurpation hypothesis’. Anim Behav 76(3): 701–708.

[3] Frederic L, Antonia D, Ram G (2009) Manipulation of host behavior by parasitic insects and insect parasites. Annu Rev Entomol 54: 189–207. 19067631 10.1146/annurev.ento.54.110807.090556

[4] Moore J (2002) *Parasites and the behavior of animals* (Oxford Univ. Press, Oxford, UK).

[5] Thomas F, Adamo S, Moore J (2005) Parasitic manipulation: where are we and where should we go? Behav Proc 68(3): 185–199. 10.1016/j.beproc.2004.06.010 15792688

[6] Adamo S (2012) In *Host manipulation by parasites*, eds Hughes PD, Brodeur J, Thomas F, (Oxford University Press, Oxford, UK), 36–51.

[7] Brodeur J, Boivin G (2004) Functional ecology of immature parasitoids. Annu Rev Entomol 49: 27–49. 14651455 10.1146/annurev.ento.49.061703.153618

[8] Seyahooei MA, Kraaijeveld-Smit FJL, Kraaijeveld K, Crooijmans JBM, Van Dooren TJM, et al (2009) Closely related parasitoids induce different pupation and foraging responses in *Drosophila* larvae. Oikos 118(8): 1148–1157.

[9] Maure F, Daoust PS, Brodeur J, Mitta G, Thomas F (2013) Diversity and evolution of bodyguard manipulation. J Exp Biol 216(5): 36–42. 23225865 10.1242/jeb.073130

[10] Brodeur J, McNeil JN (1989) Seasonal microhabitat selection by an endoparasitoid through adaptive modification of host behavior. Science 244(4901): 226–228. 17835354 10.1126/science.244.4901.226

[11] Brodeur J, McNeil JN (1992) Host behaviour modification by the endoparasitoid *Aphidius nigripes*: a strategy to reduce hyperparasitism. Ecol Entomol 17(2): 97–144.

[12] Brodeur J, Vet LEM (1994) Usurpation of host behavior by a parasitic wasp. Anim Behav 48(1): 187–192.

[13] Tanaka S, Ohsaki N (2006) Behavioral manipulation of host caterpillars by the primary parasitoid wasp *Cotesia glomerata* (L.) to construct defensive webs against hyperparastism. Ecol Res 21(4): 570–577.

[14] Grosman AH, Janssen A, de Brito EF, Cordeiro EG, Colares F, et al (2008) Parasitoid increases survival of its pupae by inducing host to fight predators. PLoS ONE 3(6): e2276. 18523578 10.1371/journal.pone.0002276 PMC2386968

[15] Janssen A, Grosman AH, Corderio EG, de Brito EF, Fonseca J, et al (2010) Context-dependent fitness effects of behavioral manipulation by a parasitoid. Behav Ecol 21(1): 33–36.

[16] Askew RR (1975) In *Evolutionary strategies of parasitic insects and mites*, ed Price PW, (Plenum Press, New York, USA), 130–153.

[17] Ito M, Hijii N (2005) Relationships among abundance of galls, survivorship, and mortality factors in a cynipid wasp, *Andricus moriokae* (Hymenoptera: Cynipidae) J For Res. 9(4): 355–359.

[18] Langellotto GA, Rosenheim JA, Williams MR (2006) Assessing trophic interactions in a guild of primary parasitoids and facultative hyperparasitoids: stable isotope analysis. Oecologia 150(2): 291–299. 16896765 10.1007/s00442-006-0514-0

[19] Sunose T (1984) Parasitoid complex of the euonymus gall midge *Masakimyia pustulae* Yukawa et Sunose (Diptera, Cecidomyiidae) in Japan. Kontyû 52(4): 557–564.

[20] Cornell HV (1983) The secondary chemistry and complex morphology of galls formed by the Cynipidae (Hymenoptera): why and how? Am Mid Nat 110(2): 225–234.

[21] Price PW, Fernandes GW, Waring GL (1987) Adaptive nature of insect galls. Environ Entomol 16(1): 15–24.

[22] Stone GN, Schönrogge K (2003) The adaptive significance of insect gall morphology. Trends Ecol Evol 18(10): 512–522.

[23] Askew RR (1961) The biology of the British species of the genus *Olynx förster* (Hymenoptera: Eulophidae), with a note on seasonal colour forms in the chalcidoidea. Proc R Entomol Soc Lond A 36: 103–112.

[24] Yukawa J, Masuda H. (1996) *Insect and Mite Galls of Japan in Colors* (Zenkoku Nôson Kyôiku Kyôkai, Tokyo, Japan).

[25] Paik JC, Yukawa J, Uechi N, Sato S (2004) Gall-inducing species of the family Cecidomyiidae (Diptera) recorded from the Korean Peninsula and surrounding islands, in comparison with the gall-midge fauna of Japan. Esakia 44: 57–66.

[26] Yukawa J, Sunose T (1976) Description of a new gall midge (Diptera, Cecidomyiidae) on Euonymus, with notes on its bionomics. Kontyû 44(2): 159–168.

[27] Sunose T (1985) Geographical distribution of two types of *Masakimyia pustulae* Yukawa & Sunose (Diptera, Cecidomyiidae) and reproductive isolation between them by parasitoid. Kontyû 53(4): 677–689.

[28] Maeda N, Sato S, Yukawa J (1982) Polymodal emergence pattern of the machilus leaf gall midge, *Daphnephila machilicola* Yukawa (Diptera, Cecidomyiidae). Kontyû 50(1): 44–50.

[29] Yukawa J (1983) Arthropod community centered upon the neolitsea leaf gall midge, *Pseudasphondylia neolitseae* Yukawa (Diptera, Cecidomyiidae) and its host plant, *Neolitsea sericea* (Blume) Koidz. (Lauraceae). Mem Fac Agr Kagoshima Univ 19: 89–96.

[30] Sunose T (1985) Population regulation of the euonymus gall midge *Masakimyia pustulae* Yukawa and Sunose (Diptera: Cecidomyiidae) by hymenopterous parasitoids. Res Popul Ecol 27(2): 287–300.

[31] Tabuchi K, Amano H (2004) Impact of differential parasitoid attack on the number of chambers in multilocular galls of two closely related gall midges (Diptera: Cecidomyiidae). Evol Ecol Res 6(5): 695–707.

[32] R Development Core Team (2010) R version 2. 12. 1: a language and environment for statistical computing. (R Foundation for Statistical Computing, Vinnea, Austria) Available: http://www.R-project.org. Accessed 2011 February 8.

[33] Holm S (1979) A simple sequentially rejective multiple test procedure. Scand J Stat 6(2): 65–70.

[34] Buhl PN (2006) Key to *Platygaster* (Hymenoptera: Platygastridae) from Denmark, with descriptions of new species. Steenstrupia 29(2): 127–167.

[35] Marchosky JR, Craig PT (2004) Gall size-dependent survival for *Asphondylia atriplicis* (Diptera: Cecidomyiidae) on *Atriplex canescens* . Environ Entomol 33(3): 709–719.

[36] Grandgirard J, Poinsot D, Krespi L, Nénon JP, Cortesero AM (2002) Costs of secondary parasitism in the facultative hyperparasitoid *Pachycrepoideus dubius*: does host size matter? Entomol Exp Appl 103(3): 239–248.

[37] Poleman ED, Reita G, Tjeerd ALS, David M, Hans MS, et al (2011) Indirect plant- mediated interactions among parasitoid larvae. Ecol Lett 14(7): 670–676. 21592275 10.1111/j.1461-0248.2011.01629.x

